# Supplementary material for: Mechanisms governing the pioneering and redistribution capabilities of the non-classical pioneer PU.1
Source: Nat Commun. 2020 Jan 21;11:402. doi: 10.1038/s41467-019-13960-2 (PMC6972792; doi:10.1038/s41467-019-13960-2)
Supplement: Supplementary file 7 — Source data [file 41467_2019_13960_MOESM7_ESM.zip › Source_Data/FigureS2/FigureS2B_MotifScanOutput/KmeansCluster6/homerResults/motif9.similar.html]

motif9

## Information for motif9

A
C
G
T
A
G
T
C
G
T
C
A
A
G
C
T
C
T
A
G
T
G
C
A
A
C
T
G
C
T
G
A
C
G
T
A
A
T
G
C
T
G
C
A
C
T
A
G
G
T
A
C
C
G
T
A
  
Reverse Opposite:  

A
C
G
T
A
C
T
G
A
G
T
C
A
C
G
T
T
A
C
G
G
A
C
T
A
G
C
T
A
G
T
C
A
C
G
T
A
G
T
C
C
T
G
A
C
A
G
T
A
C
T
G
G
T
C
A
  

|  |  |
| --- | --- |
| p-value: | 1e-75 |
| log p-value: | -1.737e+02 |
| Information Content per bp: | 1.784 |
| Number of Target Sequences with motif | 130.0 |
| Percentage of Target Sequences with motif | 3.95% |
| Number of Background Sequences with motif | 137.3 |
| Percentage of Background Sequences with motif | 0.32% |
| Average Position of motif in Targets | 110.5 +/- 52.6bp |
| Average Position of motif in Background | 95.8 +/- 58.8bp |
| Strand Bias (log2 ratio + to - strand density) | 0.5 |
| Multiplicity (# of sites on avg that occur together) | 1.00 |
| Motif File: | file (matrix) reverse opposite |

### Similar de novo motifs found

|  |  |  |  |  |  |  |  |
| --- | --- | --- | --- | --- | --- | --- | --- |
| Rank | Match Score | Redundant Motif | P-value | log P-value | % of Targets | % of Background | Motif file |
| 1 | 0.926 | C A G T G A T C C G T A A G T C T C A G C G T A A C T G C G T A T C G A G T A C C G T A A C T G A G T C | 1e-63 | -146.296799 | 3.04% | 0.20% | motif file (matrix) |
| 2 | 0.782 | A C G T C T G A A C G T T G A C G T C A G C A T C T A G C G T A C T A G C T G A T C G A G T A C C G T A A C T G | 1e-62 | -144.534248 | 5.01% | 0.82% | motif file (matrix) |
| 3 | 0.836 | G T A C C T A G G T C A A C T G C G T A C T G A A G T C C G T A A C T G A G T C C G T A | 1e-49 | -114.270914 | 3.74% | 0.56% | motif file (matrix) |
| 4 | 0.777 | T C G A A C T G C G T A C G T A A G T C C G T A A C T G A G T C | 1e-45 | -105.289282 | 11.90% | 5.19% | motif file (matrix) |
| 5 | 0.811 | C G T A A G C T T A G C G T C A C G A T A C T G C T G A C T A G C T G A T C G A T A G C C G T A A C T G | 1e-45 | -104.641942 | 3.25% | 0.46% | motif file (matrix) |
| 6 | 0.849 | G T C A A C T G C G T A C G T A A G T C G T C A A C T G A G T C C T G A | 1e-35 | -81.613525 | 4.13% | 1.02% | motif file (matrix) |
